# Prevention of Trench-Foot (Local Frigorism)

**Published:** 1918-02

**Authors:** 


**Prevention of Trench-Foot (Local Frigorism).** Abs. of an Editorial in the *Lancet*, Dec. 11, 1915, p. 1304.

The Editor reviews the progress made, up to the date of writing, especially in the prophylaxis of the disease. He calls attention especially to the work of Professor Sheridan Delépine[1], whose experiments, performed on himself, proved that while a thick wool covering retards the loss of heat from the feet, even when the covered part is plunged into the water, this retardation is not sufficient to prevent frigorism. He found, on the other hand, that a very thin layer of warm dry air between the skin and the cooling medium was sufficient to reduce the loss of heat to such an extent

1. *Lancet*, Feb. 6, 1915, and *Journal of the Royal Army Medical Corps*, for May, 1915.

that the amount of heat brought to the part by the circulating blood then sufficed to compensate for the loss. A very thin waterproof covering used in combination with a woolen layer, was found sufficient to secure a layer of warm dry air next to the skin.

The Editor also calls attention to the conclusion reached by Smith, Ritchie, and Dawson (See p. 261) that trench frost-bite is essentially similar to the stages of real frost-bite before actual necrosis has occurred. All of these writers are agreed that the chief causal factor in the usual form of trench-feet, is the loss of heat in the foot due to escape through moist shoes and wrappings which provide no layer of dry air to retain the heat. Professor Delépine also proved by experimentation, that a man standing knee-deep in cold water, without a protecting layer of dry air, might in the course of 24 hours loose as many heat calories as were developed by his food.

Similarly Behr, Webb, and Jenkins had proposed a waterproof protection for the feet in the form of a wader-footed stocking, which was perfectly water-tight, and which could be worn comfortably with the service boot. It was tried out experimentally with success, and 10,000 such wader-stockings were supplied by the government to the men at the front.

The Editor mentions favorably Professor Delépine's proposal that waterproof bags similar in shape to fishing boots, but made of oiled silk on account of its cheapness, softness, adaptability, and ease of repair if damaged, should be worn by the men in the trenches. This can be put on over a thick sock and one or more pairs of drawers. A thin sock may then be drawn over it, and the whole inserted easily in the usual army boot.

Sir R. Douglas Powell has suggested that a grease with a very low hygroscopic power, and therefore a bad conductor of heat, should be applied to the soldiers' feet. At his suggestion, Messrs. Squire and Sons had produced a cheap ointment with an absorbing power for water of only 5 o/o. This preparation might be used in addition to the waterproof stockings.

The Editor condemns absolutely the wearing of puttees during the service in the trenches. He states that the puttee is known to contract 12 o/o when wet, and is therefore calculated to obstruct the circulation. He concludes : " We earnestly hope that the puttee has really been condemned for trench use, in spite of some sacrifice in appearance. It is not worn by the French soldier, in whom the incidence of trench-foot has been notably less than in the British.... We trust that sufficient waterproof material is available to provide thigh stockings for every soldier at the front. "

